# Self-Perceived Problematic Use of Online Pornography Is Linked to Clinically Relevant Levels of Psychological Distress and Psychopathological Symptoms

**DOI:** 10.1007/s10508-021-02101-w

**Published:** 2021-11-17

**Authors:** Manuel Mennig, Sophia Tennie, Antonia Barke

**Affiliations:** 1 Department of Clinical Psychology and Psychotherapy, Philipps-Universität Marburg, Gutenbergstrasse 18, 35032 Marburg, Germany; 2 Department of Clinical and Biological Psychology, Catholic University of Eichstaett-Ingolstadt, Ingolstadt, Germany

**Keywords:** Cybersex addiction, Psychological distress, Internet, Pornography

## Abstract

Online pornography is a widespread Internet application. As with other Internet applications, in some cases its use can become problematic. First indications point to a link between problematic use of online pornography and psychological distress and general functional impairment. However, to date, there are no standardized criteria for assessing problematic use of online pornography. In this study, we used the Online Pornography Disorder Questionnaire (OPDQ)—an instrument which adapted the official criteria for Internet Gaming Disorder to online pornography—to measure problematic use and investigated to what extent consumers with a self-perceived problematic use of online pornography differed from casual users with regard to their psychological distress. An online sample of German adult visitors to a popular casual dating site completed the OPDQ, the Brief Symptom Inventory (BSI), and provided information on their online pornography use (*n* = 1539; 72.6% male; 31.43 ± 11.96 years). *T*-scores for the BSI were calculated and independent *t*-tests were conducted to compare casual users with consumers with a self-perceived problematic use of online pornography. Of the users, 5.9% fulfilled the criteria for problematic use. This group consumed online pornography for longer amounts of time and showed higher levels of psychological distress (Hedges’ *g* from 0.75 to 1.21). The *T*-scores of users with self-perceived problematic online pornography use reached clinically relevant levels on all subscales. Overall, the results of the study indicate that self-perceived problematic use of online pornography seems to be linked to severe psychological distress that may warrant clinical attention.

## Introduction

Since the inclusion of Internet Gaming Disorder (IGD) in the fifth version of the Diagnostic and Statistical Manual of Mental Disorders (DSM-5) as a “condition for further study” (American Psychiatric Association, 2013), there has been a growing interest in various specific areas of Internet use that may become clinically relevant. One of these areas is the excessive consumption of online pornography (OP). Online pornography is one of the most used Internet applications and its consumption is a widespread phenomenon in Western society (Short et al., 2012). This is reflected by the fact that one of the most popular OP websites—Pornhub—is ranked the eighth most visited website worldwide, with 33.5 billion visits in 2018 (Pornhub, 2018; SimilarWeb, 2018). By way of illustration, this corresponds to about 92 million hits per day, which is roughly equivalent to the combined population of Australia, Canada, and Venezuela. Overall, there are four online pornography websites in the top 20 of the most visited websites worldwide (SimilarWeb, 2018).

For most users, the consumption of OP is unproblematic and even some positive effects have even been observed (Litras et al., 2015; McKee, 2007; Short et al., 2012). Nonetheless, for a small proportion of users the consumption of OP appears to become problematic (Short et al., 2012; Wéry & Billieux, 2017). Since there are no standardized criteria for defining problematic use, there is as yet no agreement among researchers as to what exactly corresponds to problematic use (Duffy et al., 2016; Sniewski et al., 2018). There is, however, a consensus that excessive use of OP can become a problem and, in their systematic review, Duffy et al. (2016) identified three recurring characteristics in definitions of problematic use: excessive use of OP, negative consequences or functional impairments, and reduced control over the use of OP.

Due to the inconsistent diagnostic criteria and the resulting multitude of different diagnostic tools, it is difficult to provide accurate information on the prevalence of a problematic use of OP. In addition, most of the studies used convenience samples to investigate the prevalence of problematic use (de Alarcón et al., 2019). Therefore, the reported prevalence rates vary between 0.7 and 9.8% (Ballester-Arnal et al., 2017; Bőthe et al., 2018; Najavits et al., 2014; Ross et al., 2012). At present, only the study by Rissel et al. (2017) analyzed a nationally representative sample (Australia: *n* = 20,094). They found prevalence rates of 1.2% for women and 4.4% for men. In most of the studies, problematic use is three to five times more frequent in men than in women (Wéry & Billieux, 2017). Moreover, the problematic use of online pornography seems to be more common among young, well-educated single men (Ballester-Arnal et al., 2014; de Alarcón et al., 2019; Wéry & Billieux, 2017). However, it should be noted that these findings may be partly due to the respective samples (= student samples) that were analyzed and cannot be generalized (Wéry & Billieux, 2017).

Problematic use of OP has been linked to a number of different problems. Consumers with problematic use of OP report emotional difficulties (Allen et al., 2017; Short et al., 2012), such as feelings of shame and guilt, as well as increased feelings of inadequacy, worry, and aggression (Duffy et al., 2016; Kingston et al., 2008; Sniewski et al., 2018). Furthermore, problematic use correlates with relationship and interpersonal problems, such as disputes, lying, or social isolation (Allen et al., 2017; Duffy et al., 2016; Levin et al., 2012; Wéry & Billieux, 2017). In addition, the problematic use of OP is also associated with academic or professional problems (Duffy et al., 2016; Ross et al., 2012; Wéry & Billieux, 2017). Moreover, there seems to be an association between problematic OP use and psychopathological symptoms. These include symptoms of depression, anxiety, stress, loss of concentration, lower self-esteem, as well as reduced physical and psychological well-being (Duffy et al., 2016; Kor et al., 2014; Sniewski et al., 2018; Young, 2008). This is also corroborated by studies in the area of compulsive sexual behavior that focused on the problematic use of online pornography: They also reported that users who met the criteria for compulsive sexual behavior often suffered from psychiatric disorders such as mood, anxiety, substance-use, impulse-control, or personality disorders (Kraus et al., 2015, 2016; Raymond et al., 2003). Grubbs et al., (2015a) conducted a longitudinal study with a one-year follow-up in which they examined the relationship between problematic OP use and psychological distress. Their findings suggest that problematic use of OP is a predictor for psychological distress. This link emphasizes the clinical relevance of problematic use of OP. However, two major limitations must be considered when interpreting these previous findings. First, these studies are—with one exception—cross-sectional studies, so it is not appropriate to draw any conclusions regarding the causal relationships. OP may be the cause of the associated problem, but of course it is just as possible that problematic OP use is a coping strategy for dealing with psychological distress and/or that the relationship between problematic OP use and psychological distress is mediated by other variables (Wéry et al., 2020) or goes back to a common cause. Perry (2018) was able to show that even low usage time of OP is associated with depressive symptoms if the users experience moral incongruence. For users who do not experience moral incongruence, only very high usage times were associated with depressive symptoms, which could actually indicate reverse causality, i.e., the problematic use of OP as a coping strategy. Second, the number of studies that investigated the relationship of problematic use of OP with psychological distress is overall still very limited and studies using more strongly standardized assessments are needed.

Therefore, the aim of this study is to examine in more detail to what extent consumers with a self-perceived problematic use of OP differ from casual users, especially with regard to their psychological distress. As mentioned above, no standardized criteria currently exist to identify a problematic use of OP. Thus, in this study we use a questionnaire that utilizes the official DSM-5 criteria for IGD to assess problematic use of online pornography—the Online Pornography Disorder Questionnaire (OPDQ; (Mennig et al., 2020; Petry et al., 2014). As this questionnaire is a self-report instrument and the assessment of the severity of the problem left exclusively to the respondents, we consider the term "self-perceived problematic OP use" (SPP-OP use) to be more appropriate than "problematic OP use" and will therefore use this term for our study. At this point, it could be argued that IGD and a SPP-OP use are not the same and, therefore, the use of the same criteria is not applicable. This is a serious question that needs further research. We suggest using the IGD criteria as a starting point for such research for the following reasons. Many researchers criticize that the DSM-5 diagnosis "Internet Gaming Disorder" is too specific and instead advocate using a general concept of "problematic internet use" that covers the problematic use of all Internet applications (including OP) (Block, 2008; Potenza, 2014; Love et al., 2015). However, regarding the particular case of the problematic use of OP, many researchers argue that it should be classified as a specific Internet use disorder (Brand et al., 2016; Garcia & Thibaut, 2010; Kuss et al., 2014; Laier & Brand, 2014). This proposal seems reasonable, since there are major etiological parallels between the problematic use of computer games (IGD) and online pornography. Both behaviors are often classified as behavioral addictions and in their I-PACE model Brand et al. (2016) postulate, that the mechanisms involved in the emergence and maintenance of problematic use of Internet applications—be it computer games or online pornography—are very much alike. Therefore, it seems quite plausible to consider the problematic use of OP in the framework of problematic Internet use and accordingly use criteria that have already been well investigated in the context of another specific Internet use disorder (IGD). Furthermore, the fact that the IGD criteria also correspond well with the features defining a problematic use of OP extracted by Duffy and co-workers in their systematic review (2016) also supports the application of the IGD criteria.

## Method

### Participants and Procedure

The data were collected via an online survey (October 2017–January 2018). The link to the questionnaire was posted to various Internet forums (e.g., reddit), Facebook groups, mailing lists, and a popular German website for casual dating (poppen.de). Participants could win one of five gift vouchers for a popular online store (value: €20 each). Participants were included if they gave informed consent, were 18 years or older, reported their native language to be German, and their OP use was at least 1% of their total online time.

The inclusion criteria were fulfilled by 2443 participants. Of these, 904 (36.27%) had to be excluded: 839 because they had missing data for the OPDQ, 9 because they had missing data for the Brief Symptom Inventory (BSI; less than 40 of 53 items), 37 because they failed to provide serious information (e.g., mean OP usage session: 72 h), eight because of comments that suggested that their data were biased (e.g., high BSI values because of the recent death of a close friend, as explained in the comment section at the end of the survey), and 11 because they had an unrealistically fast answering time (2 SDs below the mean time). In the end, the data of 1539 participants were analyzed. To test for systematic dropout effects, participants who completed the OPDQ and those who terminated their participation prior to it were compared using independent *t*-tests.

Prior to beginning this study, ethics approval was obtained from the local internal review board. The participants were informed about the study; they confirmed that they were above 18 years of age and gave informed consent by clicking a consent button before they could access the survey. All data were collected anonymously.

### Measures

#### Sociodemographic Information

Information regarding sex, age, education level, as well as employment and relationship status was collected.

#### Information Regarding General and Specific Internet Use

The participants reported how much time (hours) they spend online in a typical week. In addition, they provided specific information regarding their OP use, such as what kind of OP they use and how long they use it (hours/week).

#### Problematic Use

The tendency of SPP-OP use was assessed using the OPDQ. The OPDQ is a version of the Internet Gaming Disorder Questionnaire (IGDQ; Petry et al., 2014) that was modified to assess SPP-OP use (Mennig et al., 2020) and consists of nine items, with a dichotomous response format of “no” (0) and “yes” (1). The items are modelled on the DSM-5 criteria for IGD and a total score is calculated by adding the responses (score range: 0–9). In the original IGD questionnaire, a score of ≥ 5 was defined as a cutoff above which the respondent was deemed to fulfill the DSM-5 criteria for the IGD. In order to adapt it to SPP-OP use, the references in the gaming items were replaced by references to OP. An example item is: “Do you feel that you should spend less time watching OP but are unable to cut back on the amount of time you spend watching OP?”. The psychometric evaluation indicated that this is a useful instrument for the questionnaire-based assessment of problematic use of OP (Mennig et al., 2020). The OPDG showed good internal consistency with ω_ordinal_ = 0.88. In an exploratory factor analysis, one factor was extracted and this result was validated by a confirmatory factor analysis. This finding indicates construct validity. The fact that the OPDGQ scores were highly correlated with the scores of a modified version of the Short Internet Addiction Test (original: Young, 1998; German version: Pawlikowski et al., 2013) that is designed to assess problematic Internet use, or in our case, SPP-OP use, is an indication of convergent validity. In addition, it was found that users who exceeded the cutoff for problematic use had longer periods of OP use. This finding supports the criterion validity of the instrument.

#### Brief Symptom Inventory

The validated German version of the BSI was used to assess the perceived psychological distress of the participants (Derogatis, 1993; Franke, 2000). The BSI consists of 53 statements asking about the participant's psychological functioning over the last week. The items are answered on a 5-point scale ranging from 0 (*not at all*) to 4 (*extremely*) and form nine different subscales. In addition, a global indicator of psychological distress can be calculated—i.e., global severity index (GSI). The GSI combines the number of symptoms with their intensity level. Its scores range from 0 to 4 with higher scores indicating greater distress. In the present sample, the internal consistency (Cronbach’s alpha) of the global scale was α = 0.96. The raw values of the BSI can be transformed into *T-*scores using sex-specific norms (Franke, 2000). *T*-scores (*M* = 50, SD = 10) follow a normal distribution, so that scores between 40 and 60 are considered to be average (Michel & Conrad, 1982). According to Derogatis (1993), a GSI *T*-score of ≥ 63 indicates that the distress is clinically relevant.

### Data Analysis

IBM SPSS Statistics 25 (IBM SPSS Statistics) was used for the statistical analyses. Independent *t* tests (in the case of unequal variances: Welch’s tests) were conducted to identify any differences between casual users (OPDQ score < 5) and consumers with a SPP-OP use (OPDQ score ≥ 5). These groups were compared regarding Internet usage (h/week), OP usage (h/week) and psychological distress (BSI results). The raw values of the BSI were transformed into standardized *T*-scores using the available sex-specific norm tables in order to take into account sex-specific variations in reported psychopathological symptoms (Franke, 2000). This permits comparing the BSI results in the context of a standardized *T*-distribution, which facilitates the interpretation and comparability of the results with population values. Because the group sizes of consumers with a SPP-OP use and casual users differ considerably, we report Hedges *g* (Sawilowsky, 2009) as measure of effect size. Effects of *g* = 0.20 are regarded as small, *g* = 0.50 as medium, and *g* = 0.80 as large. Because multiple comparisons were conducted, a Bonferroni–Holm correction was applied to control the family-wise error rate (Holm, 1979). To evaluate the risk of common method bias Harman's Single Factor Score was calculated (Harman, 1976; Podsakoff et al., 2003). The test is conducted by loading all relevant variables into one factor in an exploratory factor analysis and then examine the unrotated factor solution. The basic assumption of this test is that common method variance is present when the single factor explains more than 50% of the variance (Podsakoff et al., 2003).

## Results

### Descriptive Statistics

The final sample consisted of 1539 German-speaking pornography users (72.6% male) between 18 and 76 years (31.43 ± 12 years). Most of the participants completed second level education (42.3%) or a university degree (35.8%). About half of the participants were in a relationship (47.7%). The most popular form of OP was videos (54.5%), followed by pictures (35.8%). For details see Table 1.

**Table 1:** Demographic data of the participants

	*M* or *n*	SD or %
Age	31.43	11.96
Sex	1118^a^|421^b^	72.6^a^|27.4^b^
Internet usage (h/week)	22.31	15.56
Online Pornography usage (h/week)	3.17	5.11
Relationship status		
Single	717	46.6
In a relationship	735	47.7
No information provided	87	5.7
Education		
No school certificate	3	0.2
Secondary school certificate	334	21.7
A-Levels	651	42.3
University student	551	35.8
Type of online pornography		
Videos	838	54.5
Pictures	551	35.8
Webcam	145	9.4
Other	5	0.3

*n* = 1539

^a^Men

^b^Women

### Dropout Comparison

Participants who stopped their participation prior to the OPDQ were younger [*M* = 31.5 ± 11.7 years vs. *M* = 32.7 ± 12.5 years, *d* = 0.09; (*t *(1856) = 1.97, *p* < .05)] and had higher OP usage times [*M* = 4.96 ± 2.28 h vs. *M* = 4.06 ± 2.10 h, *d* = 0.11; (*t *(893) = 2.12, *p* < .05)] than those who completed it.

### Comparison of Casual Users and Consumers With a SPP-OP Use

The participants had a mean OPDQ score of 1.4 ± 1.7, with 91 (5.9%) participants reaching an OPDQ score of five points or more (= SPP-OP use); most of these were male (*n* = 80; 87.9%). For men, the prevalence of SPP-OP use was 7.15%, for women 2.61% (χ^2^ (1) = 11.35, *p* < .001). There were no significant differences regarding age (*t *(1537) = 1.04, *p* = .29), education (χ^2^ (6) = 2.24, *p* = .89), and relationship status (χ^2^ (3) = 2.39, *p* = .49).

#### Internet and OP Use

Consumers with SPP-OP use spent more time on the Internet in general (*M* = 24.46 h ± 18.08 vs. *M* = 22.05 h ± 15.37) as well as on OP (*M* = 7.85 h ± 10.05 vs. *M* = 2.89 h ± 4.49). Both differences were significant [Internet use: *t *(98.35) = 2.28, *p* < .05, *g* = 0.28 | OP use: *t *(92.27) = 4.42, *p* < .001, *g* = 0.94].

#### Psychological Distress

**Fig. 1:**
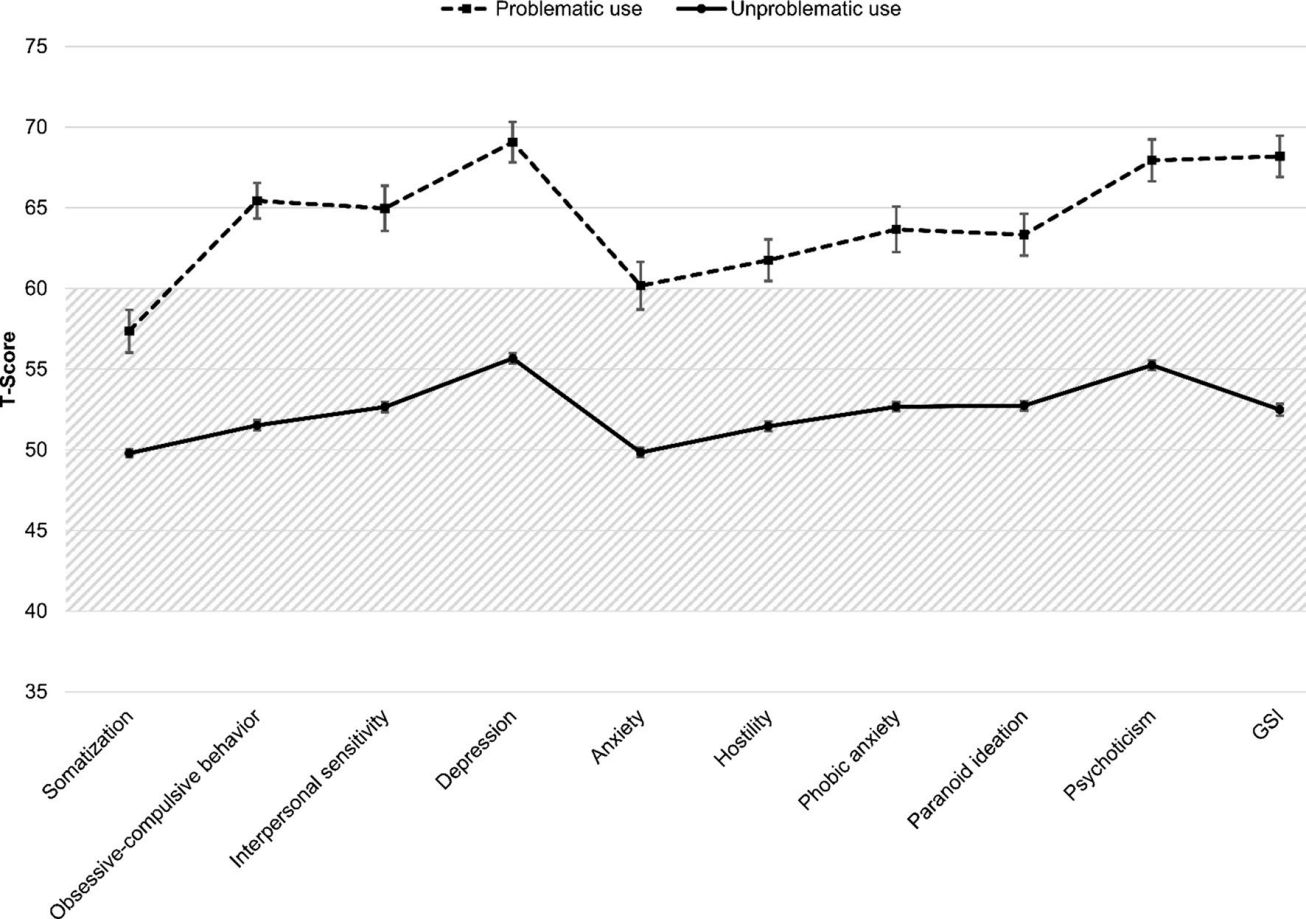
Psychological distress of consumers with problematic use of OP and casual users (all differences are significant, *p* < .01; gray hatching indicates the area where a test result is considered average; error bars (standard error) for casual use are in the order of graph point size)

Consumers with SPP-OP use scored significantly higher on every BSI subscale (*p* < .01 in all cases). They showed higher levels of somatization (*t *(97.09) = 5.59*, g* = 0.75), obsessive–compulsive behavior (*t *(104.86) = 12.16*, g* = 1.21), interpersonal sensitivity (*t *(1537) = 9.19*, g* = 0.99), depression (*t *(1537) = 10.18*, g* = 1.10), anxiety (*t *(96.77) = 6.87*, g* = 0.94), hostility (*t *(1537) = 8.29, *g* = 0.89), phobic anxiety (*t *(96.79) = 7.59, *g* = 1.04), paranoid ideation (*t *(1537) = 8.67, *g* = 0.94), and psychoticism (*t *(1537) = 10.18, *g* = 1.10), resulting in an overall higher level of psychological distress (*t *(1537) = 10.32, *g* = 1.12). See Fig. 1.

#### Harman's Single Factor Score

The unrotated exploratory factor analysis with all relevant variables loading on one factor explained 31.4% of total variance, thus speaking against common method bias.

## Discussion

In the present study, a sample of 1539 OP users was examined regarding SPP-OP use, general Internet usage behavior, sociodemographic features, and psychological distress.

The prevalence of SPP-OP use was 5.9%. Although comparing prevalence rates is difficult due to the different diagnostic instruments that are used, this result is comparable to some other studies. Daneback et al. (2006) reported a prevalence rate of 5.6% in their study of Swedish adults. In a study on Hungarian adults, 3.6% of the participating pornography users belonged to the “at-risk” group, which roughly corresponds to a problematic use (Bőthe et al., 2018). With its design, the present study was not a prevalence study. Participants were recruited deliberately so as to include a good number of self-perceived problematic users by using casual dating site which may be visited more frequently by persons also more likely to endorse problematic levels of OP. SPP-OP use was much more frequent in men than in women. This finding is well reported and found in all related studies (e.g., Daneback et al., 2006; Giordano & Cashwell, 2017; Ross et al., 2012). Contrary to some other studies, we found no differences between consumers with a SPP-OP use and casual users regarding age, education, and relationship status (Ballester-Arnal et al., 2014; Daneback et al., 2006; Ross et al., 2012).

Participants with SPP-OP use not only spent more time online in general, but consumed more OP in particular. This is in line with the results of Bőthe et al. (2018) (*r* = .14, *p* < .1), Grubbs and et al., (2015b) (*r* = .19, *p* < .01) and Brand et al. (2011) (*r* = .20, *p* > .05) who all found small positive correlations between usage time and problematic use of OP, although it depends on sample size whether they reach significance. Therefore, defining a problematic use of OP only on the basis of usage time of OP is not appropriate.

By far the largest difference between consumers with a SPP-OP use and casual users was found with regard to their psychological distress. Participants with SPP-OP use scored higher on every subscale of the BSI, indicating that their level of psychological distress was markedly higher than that of their counterparts. The most pronounced differences were found on the subscales depression, obsessive–compulsive behavior, and psychoticism. The link between SPP-OP use and depression is one of the more researched subjects in the literature and was confirmed in this study which has standardized diagnostic criteria and a larger sample (Grubbs, et al., 2015a; Philaretou et al., 2005; Wéry & Billieux, 2017). The heightened scores of participants with a SPP-OP use on the subscales obsessive–compulsive behavior and psychoticism may be influenced by differences in personality factors that have been linked to problematic OP use. Previous studies reported an association between problematic Internet use (including OP) and higher levels of impulsivity and neuroticism (Antons & Brand, 2018; Hardie & Tee, 2007; Müller et al., 2014a, 2014b; Wang et al., 2015). These personality traits have been reported to be related to the BSI subscales obsessive–compulsive behavior (impulsivity) and psychoticism (neuroticism) (Grassi et al., 2015; Loutsiou-Ladd et al., 2008). This study’s confirmation that consumers with SPP-OP use show an overall higher level of psychological distress further corroborates existing reports. Grubbs and colleagues (Grubbs et al., 2015a, 2015b) conducted two studies examining the relationship between self-judged addiction to OP and psychological distress. In both studies, they found that higher levels of perceived addiction to OP were linked to psychological distress. In their longitudinal study (Grubbs et al., 2015a), the relationship remained significant even when they controlled for other variables like baseline psychological distress or usage time of OP. In their analyses of a sample of treatment seekers for Internet addiction (which included problematic use of OP), Müller et al., (2014a, 2014b) compared participants that met the criteria for Internet addiction and those who did not regarding their psychological distress. They also found that Internet addiction was associated with higher levels of psychological distress (GSI: 0.83 vs 0.35, *p* < .001). In contrast to our study, Müller et al., (2014a, 2014b) analyzed a broad sample of patients with Internet addiction (which also included online gaming or social networking sites). Because we only focused on users of OP, the results of our study allow us to conclude specifically with regard to SPP-OP use. Studies in the area of research on sexual addiction or compulsive sexual behavior likewise found a relationship between a problematic use of online pornography and increased psychological distress. In an online study, Kor et al. (2014) found that the scores of a questionnaire on the problematic use of online pornography were positively correlated with psychological distress. They also used the BSI to capture psychological distress of the participants and—in line with our results—found correlations between *r* = .18 (somatization) and *r* = .27 (psychoticism). In another interesting study with a clinical sample, Kraus et al. (2015) examined 103 men seeking treatment for compulsive pornography use and/or sexual promiscuity. They found that the majority of participants not only had a problem with the use of online pornography, but also met the criteria of the following psychiatric disorders: mood (71%), anxiety (40%), substance-use (41%), and impulse-control disorders (24%).

In the present study, participants with SPP-OP use not only had higher BSI values than casual users, but most of their results were elevated to a clinically relevant degree measured against the population norm of the BSI. The *T-*scores of their GSI as well as their results on the subscales obsessive–compulsive behavior, interpersonal sensitivity, depression, phobic anxiety, paranoid ideation, and psychoticism were ≥ 63. In particular, the GSI scores of *T* = 68 (raw value: GSI = 1.12) are remarkable, because this corresponds to a percentile rank of 96%, meaning that 96% of the norm group scored lower. Such high scores are usually only obtained by people with psychiatric disorders (Kellett et al., 2003). Wieland et al. (2012) analyzed a sample of psychiatric outpatients with intellectual disabilities. The subgroup that also met the DSM-4 criteria for a psychiatric disorder obtained a BSI total score of GSI = 1.10. By contrast, the BSI values of the casual users were all within the range of the population norm of between *T* = 40–60. This suggests that the consumption of online pornography in itself is unproblematic, whereas people with SPP-OP use were in severe psychological distress. However, since this is a cross-sectional study, we cannot make any reliable statements about the causality of the relationship. It is possible that SPP-OP use could lead to problems (e.g., social withdrawal), which may subsequently lead to psychological distress. Grubbs et al., (2015a, 2015b) conducted a longitudinal study and found that self-perceived addiction to OP predicted psychological distress. The relationship remained significant even when they controlled for other variables like baseline psychological distress or usage time of OP. These results establish a certain temporal order. Since temporal precedence is a necessary condition of causality, these findings are compatible with the view that psychological distress leads to SPP-OP use. However, it is not a sufficient condition and so no definite causal interpretation of the relationship is permissible, since other relevant, yet unmeasured third variables could account for the association. Psychological distress and SPP-OP use may both be consequences of a common cause, such as a shortcomings in self-regulatory emotional and cognitive processes, early adversity or other transdiagnostic factors (Gershon et al., 2013; Sheppes et al., 2015). In clinical experience, most often, these different causal paths coexist and interact. As already mentioned in the introduction, it is of course also conceivable that there is reverse causality. In other words, SPP-OP may be a reaction to already existing psychological distress. In this case, the SPP-OP would be a coping strategy for the psychological distress.

### Strengths and Limitations

Among the strengths of the current study are the large sample size of pornography users recruited, the determination of SPP-OP use using criteria analogous to the DSM-5 criteria for IGD, and the use of BSI *T-*scores which facilitated meaningful comparisons with population norms.

The interpretation of the results should take into account the study’s limitations, such as its cross-sectional design which precludes any causal inferences, the self-selected nature of the sample, and the exclusive use of self-report measures.

### Conclusion

Overall, the results of this study suggest that SPP-OP use is linked to severe psychological distress. We note the existence of a group suffering simultaneously from SPP-OP use and elevated psychopathological symptoms and high distress. Therefore, in the treatment setting, it might be useful to explore the use of OP, as problematic use might be a perpetuating factor for existing psychological distress and may be a serious problem, which demands awareness and, in some cases, clinical attention. When one considers that consuming OP is one of the most popular online activities engaged in by millions of people all over the world, future studies should further investigate what relationships obtain between SPP-OP use and psychological distress with experimental and longitudinal designs.
